# Characteristics of SARS‐CoV‐2 positive individuals in California from two periods during notable decline in incident infection

**DOI:** 10.1002/hsr2.384

**Published:** 2021-10-06

**Authors:** Lao‐Tzu Allan‐Blitz, Isaac Turner, Fred Hertlein, Jeffrey D. Klausner

**Affiliations:** ^1^ Division of Global Health Equity, Department of Medicine Brigham and Women's Hospital Boston Massachusetts USA; ^2^ Curative Inc. San Dimas California USA; ^3^ Department of Population and Public Health Sciences Keck School of Medicine, University of Southern California Los Angeles California USA

## 1 INTRODUCTION

**FIGURE 1:**
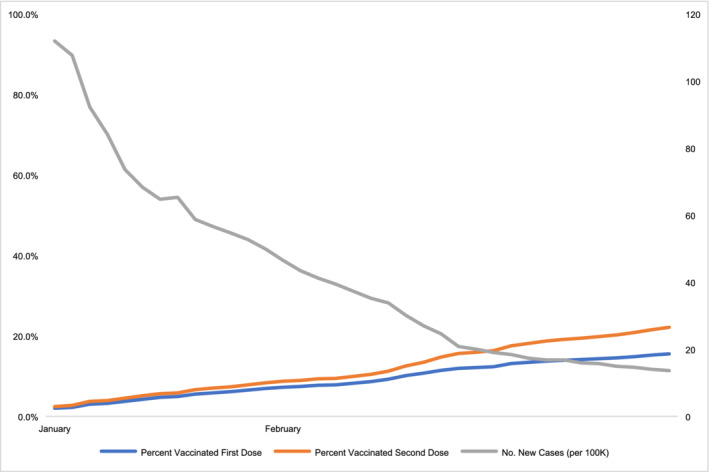
New severe acute respiratory syndrome coronavirus 2 (SARS‐CoV‐2) infections against percent vaccinated in California, January–March 2021. This figure shows percent vaccinated (by first and second dose) and new SARS‐CoV‐2 cases per 100 000 individuals in California between January and March 2021. Data extrapolated from U.S. COVID Risk & Vaccine Tracker^2^

Between February and May 2021, the weekly case rate of severe acute respiratory syndrome coronavirus 2 (SARS‐CoV‐2) declined in California from 270 to 32.9 cases per 100 000 individuals.^1^ The cause of the dramatic decline is likely multifactorial but greatly influenced by population‐level immunity due to prior infection and increasing vaccination coverage (see Figure 1).^2^

SARS‐CoV‐2, however, continues to be transmitted heterogeneously among different subsets of the population.^3^ The recent resurgence (from 17 to 173 cases per 100 000 individuals between June and August in California)^1^ and fears that SARS‐CoV‐2 variants^4^ may escape immunity warrant continued epidemic monitoring. We aimed to characterize individuals testing positive for SARS‐CoV‐2 during the period of notable decline in case rate in California to generate hypotheses for understanding shifting risk dynamics that may contribute to the current trend in viral transmission.

## 2 METHODS

We conducted a retrospective cohort study among individuals presenting for SARS‐CoV‐2 polymerase chain reaction (PCR) testing to one of 150 drive‐through publicly accessible commercial sites in California at two periods: (a) February 23‐March 3, 2021, and (b) between April 15 and 30 2021. Testing sites were located in Los Angeles, Riverside, San Mateo, Berkeley, Menlo Park, Maywood, and Rancho Mirage. As a part of the testing program, all individuals completed a confidential online survey reporting demographic and employment information, if in the last 14 days, they had been contacted by local public health authorities about a known SARS‐CoV‐2 exposure or visited any of a list of public places, as well as report of symptoms at the time of testing. The survey was completed via personal or provided smart device. All individuals were included in the analysis who had testing data available, regardless of survey completion.

We collected data on PCR results from healthcare worker‐observed self‐collected oral swab specimens, which have been shown to have a near 100% positive and negative percent agreement with clinician‐collected nasopharyngeal swabs^5^ and were processed with standard PCR methods using a modified Food and Drug Administration (FDA)‐authorized Center for Disease Control and Prevention testing protocol as has been previously reported.^6^ For that PCR assay, a cycle threshold value of 30 corresponded to approximately 3000 viral copies per mL (range 1500‐6000 copies per mL) of solution.

We then conducted a cross‐sectional descriptive analysis to determine the frequency of infection among testers and positivity ratios for the two periods based on each of the above characteristics. We stratified our analysis by Hispanic heritage to account for confounding.^7^ The Mass General Brigham institutional review board deemed the analysis of de‐identified data did not constitute human subjects' research (2020P003530). All analyses were conducted using STATA 15.1 (StataCorp, College Station, TX).

## 3 RESULTS

We analyzed 114 789 test results (see Table 1). Of 529 07 results between February 23‐March 3, 2021, 2679 (5.1%) were positive. Of 61 882 test results from April 15‐30, 1579 (2.6%) were positive. Testers included 48.0% who identified with Hispanic heritage, and 54.6% reported female sex.

**TABLE 1:** Severe acute respiratory syndrome coronavirus 2 (SARS‐CoV‐2) positivity among Hispanics and non‐Hispanic individuals from California comparing February 23 to March third and April 15 to April 30, 2021

	Hispanic ethnicity							Non‐hispanic ethnicity						
	Total		No. SARS‐CoV‐2 positive		SARS‐CoV‐2 positivity		Positivity ratio	Total		No. SARS‐CoV‐2 positive		SARS‐CoV‐2 positivity		Positivity ratio
	Feb	Apr	Feb	Apr	Feb	Apr	Feb vs Apr	Feb	Apr	Feb	Apr	Feb	Apr	Feb vs Apr
Total	24 929	30 119	1907	907	7.6%	3.0%	2.5	27 978	31 763	772	672	2.8%	2.1%	1.3
Age														
<18 years	2889	8264	341	200	11.8%	2.4%	4.9	3573	8081	90	106	2.5%	1.3%	1.9
18 to 24 years	4424	5713	281	174	6.4%	3.0%	2.1	3509	3868	109	101	3.1%	2.6%	1.2
25 to 34 years	6865	6770	442	222	6.4%	3.3%	2.0	7642	6493	199	207	2.6%	3.2%	0.8
35 to 49 years	5914	5530	419	186	7.1%	3.4%	2.1	7126	7146	163	135	2.3%	1.9%	1.2
50 to 64 years	3865	3002	345	97	8.9%	3.2%	2.8	4758	4543	162	92	3.4%	2.0%	1.7
≥65 years	970	840	79	28	8.1%	3.3%	2.4	1370	1632	49	31	3.6%	1.9%	1.9
Gender														
Female	13 764	17 110	951	462	6.9%	2.7%	2.6	14 677	17 082	350	342	2.4%	2.0%	1.2
Male	11 111	12 947	954	444	8.6%	3.4%	2.5	13 212	14 610	419	328	3.2%	2.2%	1.4
Heritage														
American Indian or Alaska Native	245	338	16	4	6.5%	1.2%	5.5	59	88	5	1	8.5%	1.1%	7.5
Black or African American	203	251	11	10	5.4%	4.0%	1.4	3322	4486	62	145	1.9%	3.2%	0.6
Native Hawaiian or Another Pacific Island	73	70	10	4	13.7%	5.7%	2.4	354	442	16	12	4.5%	2.7%	1.7
Asian	95	134	3	0	3.2%	0.0%	*	6199	6812	159	101	2.6%	1.5%	*
Multiracial	401	514	18	15	4.5%	2.9%	1.5	805	1022	17	19	2.1%	1.9%	1.1
White	5365	6756	330	195	6.2%	2.9%	2.1	14 920	16 071	429	316	2.9%	2.0%	1.5
Other/prefer not to share	16 820	20 072	1450	629	8.6%	3.1%	2.8	1346	1123	60	59	4.5%	5.3%	0.8
Employment														
Agricultural or food manufacturing worker	183	323	12	10	6.6%	3.1%	2.1	143	211	4	1	2.8%	0.5%	*
Construction worker	4	9	0	3	0.0%	33.3%	0.0	4	3	0	0	0.0%	0.0%	*
Correctional facility	41	34	5	3	12.2%	8.8%	1.4	23	28	1	1	4.3%	3.6%	1.2
Delivery or ride share	2	6	0	1	0.0%	16.7%	0.0	8	1	1	0	12.5%	0.0%	*
Disability care provider	440	451	24	25	5.5%	5.5%	1.0	500	517	11	5	2.2%	1.0%	2.3
Education	1160	3812	44	23	3.8%	0.6%	6.3	1886	3694	24	36	1.3%	1.0%	1.3
Emergency services	213	253	10	4	4.7%	1.6%	3.0	216	263	4	4	1.9%	1.5%	1.2
First responder	5	4	0	0	0.0%	0.0%	*	3	2	0	0	0.0%	0.0%	*
Food services	1847	1859	159	68	8.6%	3.7%	2.4	1317	1295	52	51	3.9%	3.9%	1.0
Government personnel	5	4	0	0	0.0%	0.0%	*	2	2	0	0	0.0%	0.0%	*
Grocery store worker	8	3	0	0	0.0%	0.0%	*	3	3	0	0	0.0%	0.0%	*
Healthcare Worker	1338	1779	62	34	4.6%	1.9%	2.4	1812	2182	37	28	2.0%	1.3%	1.6
Members of the media	0	0	0	*	*	*	*	2	1	0	0	0.0%	0.0%	*
Public transportation	198	248	10	8	5.1%	3.2%	1.6	178	255	6	4	3.4%	1.6%	2.1
Retail or manufacturing	1698	1480	141	58	8.3%	3.9%	2.1	1116	890	44	23	3.9%	2.6%	1.5
Public exposures														
Bars	136	338	0	2	0.0%	0.6%	0.0	311	625	3	11	1.0%	1.8%	0.5
Restaurants	1269	3232	13	32	1.0%	1.0%	1.0	3168	5446	17	29	0.5%	0.5%	1.0
Gas stations	2076	2908	46	21	2.2%	0.7%	3.1	3990	4750	25	39	0.6%	0.8%	0.8
Public park	1006	2704	13	24	1.3%	0.9%	1.5	3337	4877	12	18	0.4%	0.4%	*
Retail store	1785	3862	34	36	1.9%	0.9%	2.0	3739	5744	17	31	0.5%	0.5%	0.8
Grocery store	3603	6457	78	65	2.2%	1.0%	2.2	6864	9132	42	62	0.6%	0.7%	0.9
Place of work	869	1204	23	7	2.6%	0.6%	4.6	1346	1705	7	12	0.5%	0.7%	0.7
Public transit	413	954	4	9	1.0%	0.9%	1.0	863	1237	4	15	0.5%	1.2%	0.4
Place of worship	243	549	4	3	1.6%	0.5%	3.0	236	512	1	1	0.4%	0.2%	*
Known COVID contact in last 14 days														
No	17 039	24 551	952	503	5.6%	2.0%	2.7	22 315	27 490	382	349	1.7%	1.3%	1.3
Yes	5812	3917	934	400	16.1%	10.2%	1.6	3662	2980	375	315	10.2%	10.6%	1.0

Abbreviation: COVID, coronavirus disease.

In the first period, the positivity of SARS‐CoV‐2 infection among Hispanic and non‐Hispanic testers was 7.6% and 2.8%, respectively (*P‐*value<.001). In the second period, the positivity among Hispanic and non‐Hispanic testers was 3.0% and 2.0%, respectively (*P‐*value = .09). Of individuals testing positive, 1309 (48.8%) and 715 (45.3%) reported contact with a known case in the last 14 days in the first and second period, respectively.

Among Hispanic testers during the first period, we found a high positivity of infection among children (11.8%) and those who reported mixed heritage (6.5%). We found consistently elevated positivity through both periods among individuals reporting any known exposure in the past 14 days (16.1% and 10.2%, respectively), individuals reporting employment as disability care providers (5.5% and 5.5%, respectively), food service providers (8.6% and 3.7%, respectively), and employment in retail or manufacturing (8.3% and 3.9%, respectively).

Among non‐Hispanic testers, we found consistently elevated SARS‐CoV‐2 positivity among individuals reporting employment in retail or manufacturing in both periods (3.9% and 2.6%, respectively), as well as among testers reporting any known exposure in the past 14 days (10.2% and 10.6%, respectively).

## 4 DISCUSSION

We evaluated SARS‐CoV‐2 positivity and potential exposures among those presenting for testing during two periods in California, identifying notable positivity among testers of Hispanic heritage and those reporting a recent known exposure. The current trends in SARS‐CoV‐2 case rates across the United States are again increasing.^1^ An epidemiologic understanding of those most at risk for infection is essential as the transmission dynamics shift, in order to guide future prevention efforts.

We found high test positivity among Hispanic testers and among children specifically during the first period, supporting the key role of within household transmission.^8^ Because schools were mostly closed during the initial period in California, and the reopening of schools predominantly overlapped with the second observation period, it is unlikely that school attendance is contributing to the continued spread of infection. Household crowding is more common among Hispanic communities,^9^ likely contributing to substantial intrafamilial transmission.

The disparities among testers identifying as individuals of minority heritage are consistent with prior studies,^7^, ^10^, ^11^ likely reflecting structural inequities continuing to put certain populations at increased risk of exposure due to inadequate protections. Differences in types of employment may play a key role such disparities.^12^ Our findings suggest that prevention strategies may benefit from focusing on businesses employing food service workers and disability care providers. Disability care providers are a particularly important population as the morbidity and mortality of SARS‐CoV‐2 infection are substantial among individuals of long‐term care facilities.^13^ Thus, we encourage requiring vaccination among such employment categories. Further research is still needed to both address the underlying structural inequities and clarify the specific exposures within different sub‐populations that contribute to sustained transmission.

Nearly half of infections in our testing population in both periods were among individuals who reported recent contact with someone known to be infected with SARS‐CoV‐2. Thus, contact tracing efforts, perhaps now more than ever, are essential for identifying and isolating remaining cases and testing and quarantine of those exposed.^14^ Incorporation of detailed exposure reporting at testing centers may complement contact tracing efforts and facilitate real‐time monitoring of the variations in risk exposures; however, such data must be collected fromboth infected and uninfected persons.^15^ Additionally, continuing to encourage testing and vaccination among individuals with a known exposure will be essential.

Our study had several limitations. First, we analyzed laboratory‐based data and could not account for individuals with repeat testing. Second, we were unable to collect detailed socioeconomic data in order to control for confounding factors. Data collection was also incomplete for several fields making further statistical analyses and modeling not possible. Thus, this study is hypothesis generating. The strengths of our study were the very large sample size, thus improving precision of our results, the unbiased collection of exposure data—when reported—prior to receiving testing results, and the inclusion of numerous testing sites across California, improving the generalizability of our results.

## 5 CONCLUSIONS

We report SARS‐CoV‐2 positivity by ethnic heritage in California from two observation periods. We found notable SARS‐CoV‐2 positivity among Hispanic testers, testers with a known recent exposure, and variations in positivity by type of employment. Those findings are important because of the evolving epidemiology of those at risk, thus providing groundwork for future research and potentially informing public health strategies.

## ACKNOWLEDGEMNTS

The authors would like to acknowledge the cities of Los Angeles, Riverside, San Mateo, Berkeley, Menlo Park, Maywood, and Rancho Mirage.

## FUNDING

Supported in part by a gift to the Keck School of Medicine of the University of Southern California by the W.M. Keck Foundation.

## CONFLICT OF INTEREST

Dr. Allan‐Blitz served as a consultant for Curative Inc., Fred Hertlein is an employee of Curative Inc., Isaac Turner is a Co‐Founder and Chief Information Officer of Curative Inc., and Dr. Klausner is the independent medical director of Curative Inc. The above financial relationships had no involvement on study design, data collection, data analysis, interpretation of the data, writing of the report, or decision to Thi submit for publication.

## AUTHOR CONTRIBUTIONS

Conceptualization: Lao‐Tzu Allan‐Blitz, Isaac Turner, and Jeffrey D. Klausner.

Formal analysis: Lao‐Tzu Analysis and Fred Hertlein.

Writing (original draft): Lao‐Tzu Allan‐Blitz.

Writing (review and editing): Lao‐Tzu Allan‐Blitz, Isaac Turner, Fred Hertlein, and Jeffrey D. Klausner.

All authors have read and approved the final version of the manuscript.

Lao‐Tzu Allan‐Blitz had full access to all of the data in this study and takes complete responsibility for the integrity of the data and the accuracy of the data analysis.

## TRANSPARENCY STATEMENT

Lao‐Tzu Allan‐Blitz affirms that this manuscript is an honest, accurate, and transparent account of the study being reported; that no important aspects of the study have been omitted; and that any discrepancies from the study as planned (and, if relevant, registered) have been explained.

## Data Availability

The data are available upon request.
